# Intracranial Hemorrhage From Cerebral Venous Thrombosis With Hypereosinophilia and Positive Dengue Serology in a Child: A Rare Case and Challenges in Management

**DOI:** 10.7759/cureus.64220

**Published:** 2024-07-10

**Authors:** Nguyen The Nguyen Phung, Minh Nhut Tran

**Affiliations:** 1 Department of Pediatrics, University of Medicine and Pharmacy at Ho Chi Minh City, Ho Chi Minh City, VNM; 2 Infectious Diseases Intensive Care Unit, Children's Hospital 1, Ho Chi Minh City, VNM

**Keywords:** child, pediatric, dengue, hypereosinophilia, eosinophilia, cerebral venous thrombosis, intracranial hemorrhage

## Abstract

Hypereosinophilia is a rare condition, defined as a persistent elevation of absolute eosinophil count greater than 1.5x10^9^/L and/or tissue eosinophilia. This condition can be caused by numerous different etiologies, both hematological (clonal) and non-hematological (reactive). Reactive hypereosinophilia encompasses all disorders, including infections. Patients with hypereosinophilia may experience a spectrum of clinical consequences due to multiple organ damage, including neurologic and thrombotic complications, associated with organ dysfunction and potentially life-threatening sequelae. Cerebral venous thrombosis (CVT) is the term used to describe thrombotic occlusion of veins and/or venous sinuses in the brain. This condition can occur at all ages and CVT related to hypereosinophilia is a rare disease. Diagnosis of the disease must be done quickly because thrombosis causes blockage of cerebral drainage, venous congestion, disruption of cerebrospinal fluid reabsorption, ischemic neuronal damage, cerebral edema, and hemorrhage, leading to severe neurological complications. Management of intracranial hemorrhage from CVT due to hypereosinophilia is a challenging task for clinicians, based on anticoagulation therapy, systemic corticosteroid, management of elevated intracranial pressure, and potentially progressive hemorrhage due to anticoagulant. The outcome of the patient generally relies on early detection, prompt, and appropriate treatment. In this case report, we discuss a rare case of CVT with hypereosinophilia and positive dengue serology in a child, in the context of intracranial hemorrhage, enlightening the importance of considering a personalized strategy in the management of this complex scenario.

## Introduction

Eosinophilia is defined as an absolute eosinophil count (AEC) greater than 0.5x10^9^/L and a condition in which the persistent elevation of AEC greater than 1.5x10^9^/L and/or tissue eosinophilia is called hypereosinophilia (HE) [1]. A high eosinophil count, especially greater than 1.5x10^9^/L, is always a clinically important feature that requires attention and explanation [2]. However, diagnostic evaluation of the causes of eosinophilia is a challenge for clinicians because the manifestation can be seen in a variety of diseases, both due to infectious and non-infectious causes [1,3,4]. Helminthic parasitic diseases are the most common causes of eosinophilia, meanwhile, acute bacterial and viral infections almost invariably cause eosinopenia [3,5]. There are some case reports of blood eosinophilia caused by viral infections, including herpes and human immunodeficiency virus (HIV) infection [3,5]. In tropical and subtropical countries in the Asia-Pacific and Latin America region including Vietnam, dengue infection is an endemic disease, mainly transmitted by a mosquito (Aedes aegypti). In practice, although the diagnosis of dengue infection is often based on clinical manifestations only, laboratory confirmation of clinical diagnosis may be valuable. The selection of laboratory tests that confirm the diagnosis depends on the date of illness, in which, dengue serologic tests are more available in dengue-endemic countries and are the methods of choice for diagnosis after Day 5 [6]. Increased eosinophil count can be observed in dengue cases and used as a hematological marker of the recovery phase [7,8].

Eosinophilic-related clinical manifestations are diverse and affect multiple organs, including complications of the nervous system. Cerebral venous thrombosis (CVT) is reported as a rare neurologic manifestation of HE that can occur in the early stage of the disease and is a life-threatening complication if not diagnosed and treated appropriately [9]. In a review article conducted by Yager et al., CVT itself is recognized as a relatively rare condition in pediatric patients, with an estimated incidence of 0.67 per 100,000 children per year in a Canadian Pediatric Stroke registry [10]. The incidence of intracranial hemorrhage (ICH) is about one-third among patients with CVT [11]. The presence of ICH in patients with CVT is an unfavorable outcome factor and a challenging task for clinicians and their teams in management [11].

Herein, we report a child diagnosed as CVT complicated by ICH with HE and positive dengue serology who was treated successfully. This study adds to the discussion of a rare neurological manifestation of dengue infection and the clinical decision to treat this rare stroke in pediatric patients.

## Case presentation

A 14-year-old previously healthy boy was admitted (Day 0 of admission) with fever, headache, nausea, vomiting, loss of appetite, difficulty speaking, loss of concentration, mood changes, irritability for five days, and weakness of the left arm and left leg for two days. He had a sustained fever with a body temperature of 39.5°C. He denied having any following symptoms and signs: cough, dyspnea, abdominal pain, constipation, diarrhea, and abnormal urine output during the five days before admission. He had never been hospitalized before for any problem, had no documented history of dengue infection, and no known allergies, or skin, respiratory, digestive, or musculoskeletal diseases. He has never had coronavirus disease-19 (COVID-19) and he received two COVID-19 vaccines of the Astra Zeneca 1.5 years ago. He had not traveled anywhere in the last six months. We also did not record any family history of hematological diseases, thrombosis, cancer, stroke, dyslipidemia, diabetes, or allergies.

At the time of admission, he was conscious with a Glasgow Coma Score (GCS) of 15. He was hemodynamically stable with vital signs were as follows: pulse rate was regular at 90 beats per minute and vascular tone was good, body temperature was 38oC, blood pressure was 95/60 mmHg, respiratory rate was regular at 20 breaths per minute regular with no retractions and no using of accessory muscle, oxygen saturation was 100% with no respiratory support, and capillary refill time was normal. Physical examination revealed his obesity with his weight of 65 kg and height of 157 cm, body mass index (BMI) was 26.3 (Obesity class I - The Asia-Pacific classification). The tourniquet test was positive. We did not note hepatosplenomegaly, bleeding, or signs of an allergic reaction.

**Figure 1:**
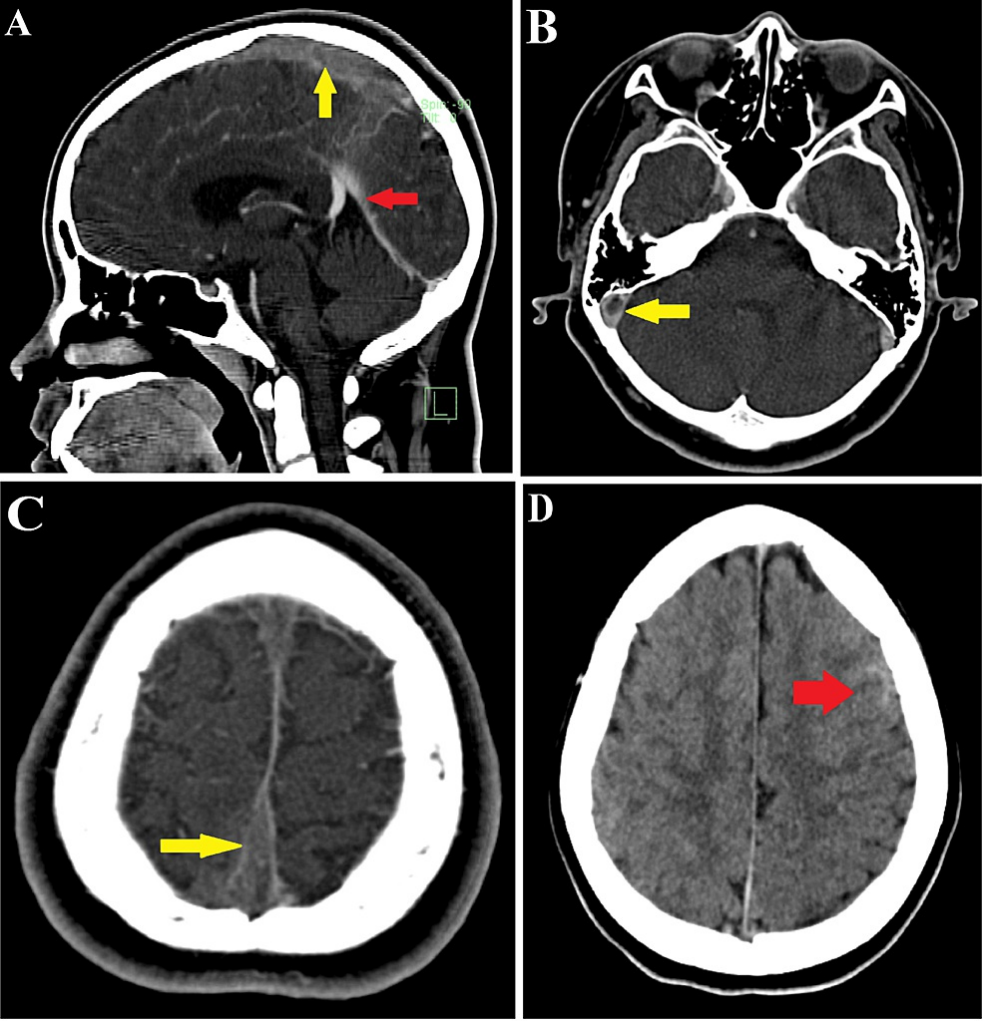
Computed tomography (CT) of the brain. (A) Thrombosis of the superior sagittal sinus (yellow arrow) and cerebral vessel with normal contrast enhancement (red arrow). (B) Thrombosis of the right transverse sinus (yellow arrow). (C) Thrombosis of the superior sagittal sinus (yellow arrow). (D) Intracranial hemorrhage in the left frontal and parietal lobe (red arrow).

The patient experienced a continuous high fever, along with progressive weakness with muscle strength was 3/5 on the left side of the body and during all movements. Cranial computed tomography (CT) was performed on Day 1 of admission, which revealed filling defects in the superior sagittal sinus and right transverse sinus representing cerebral venous sinus thrombosis, possibly cerebral edema and ICH in the left frontal lobe and left parietal lobe (Figure 1).

Approximately 24 hours after admission, he suddenly had a clonic seizure on the left side of his body, lasting about 10 minutes. During and after the seizure, he had no mental disturbance. He was transferred from the medical ward to the infectious diseases intensive care unit (ID-ICU), in the hospital for further management of CVT and seizure.

His laboratory test results within the first 48 hours showed leukocytosis - 17.01x10^3^/µL (normal range 4.0-10.5x10^3^/µL) with an AEC of 5.07x10^3^/µL, hemoglobin concentration of 12.3g/dL with a hematocrit of 37.9% and thrombocytopenia with platelets (PLT) count of 28x10^3^/µL (normal range 150-400x10^3^/µL) and rapidly decreased to 19x10^3^/µL over 12 hours after admission. The coagulation test (Day 1) showed prothrombin time (PT) of 15.6 s (reference range 12.9-16.1 s), activated partial PT (aPTT) of 26.3 s (reference range 28.0-37.9 s) and fibrinogen of 3.61 g/L (reference range 1.84-3.61 g/L), D-dimer increased to > 20 μg/mL (because the laboratory machine at our hospital cannot measure results greater than this value). C-reactive protein (CRP) was elevated (102.71 mg/L), liver damage with aspartate aminotransferase (AST) 157.6 U/L and alanine aminotransferase (ALT) 611.4 U/L (normal range of AST and ALT < 50 U/L), bilirubin (total, direct and indirect) were normal. Kidney function tests were normal: serum urea 3.16 mmol/L and serum creatinine 57.8 μmol/L, sodium 135.2 mmol/L, potassium 3.78 mmol/L, and calcium ion 1.13 mmol/L. Immunoserology tests for evaluation for etiologies of hepatitis were negative for hepatitis A immunoglobulin M antibody (HAV IgM), hepatitis B surface antigen (HBsAg) and hepatitis C virus (HCV), HIV Ab, herpes simplex virus immunoglobulin M and G (HSV - IgM and IgG) antibodies, Epstein-Barr virus Deoxyribonucleic acid (EBV DNA) and cytomegalovirus Deoxyribonucleic acid (CMV DNA). He had a lumbar puncture on Day 2 with the following normal results: 1 cell/mm^3^, cerebrospinal fluid (CSF)/blood glucose ratio of 61%, CSF lactate of 1.893 mmol/L and CSF protein of 0.489 g/L. Urinalysis did not reveal hematuria or proteinuria. Chest X-ray, cardiac Doppler ultrasound, and abdominal ultrasound were normal.

**Figure 2:**
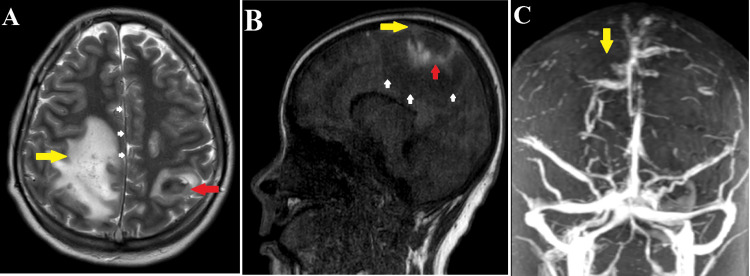
Magnetic resonance imaging (MRI) of the brain. (A) Hemorrhagic infarct in the left parietal region (red arrow) and cerebral edema in the right parietal lobe (yellow arrow), causing midline shift (white arrow). (B) Thrombosis in the superior sagittal sinus (yellow arrow) and hemorrhagic infarct (red arrow), causing cerebral edema (white arrow). (C) Magnetic resonance venogram showing non-visualization of cortical vein, suggesting thrombosis.

Before transferred to ID-ICU, he received one unit of PLT (40 mL) and used low molecular weight heparin (LMWH) 1 mg/kg subcutaneously every 12 hours. In ID-ICU, he had recurrent seizures and altered mental status with a GCS of 12, so he was intubated and received mechanical ventilation on Day 3. He was given broad-spectrum intravenous antibiotics (Meropenem and Linezolid) and acyclovir because we could not rule out sepsis and HSV. His optic nerve sheath diameters (ONSD) by ultrasound in the right eye was 7.3 mm and the left eye was 7.1 mm with papilledema, so we thought that the patient had elevated intracranial pressure (ICP). We rapidly performed management of increased ICP including hypertonic saline and mannitol infusions, hyperventilation with a target pCO2 of 30-35 mmHg. The dose of LMWH was reduced to 0.5 mg/kg every 12 hours because we thought the ICH might be getting worse. Cranial magnetic resonance imaging (MRI) was done on Day 6 which showed superior sagittal sinus thrombosis and cortical venous thrombosis in the fronto-parietal region on both hemispheres, complicating ICH in the left frontal-parietal region and right parietal lobe (Figure 2). There was an increased AEC in the complete blood count results on Day 6, while white blood cell count was 16.76x10^3^/µL (neutrophil 66.6%, lymphocyte 11.6%, monocyte 8.7%) and on the same day, there were no blast cells or abnormal cells detected in the peripheral blood smear, so we decided to use methylprednisolone 2 mg/kg/day. Procalcitonin (Day 8 after admission) was 0.30 ng/mL (normal range < 0.1 ng/mL).

Dengue IgM enzyme-linked immunosorbent assay (ELISA) was positive (Day 13). The patient was tested to evaluate hypercoagulability with the following results: lupus anticoagulant screen was positive, lupus anticoagulant confirm was weakly positive 1.21 (reference range for weakly present: 1.20-1.49), other laboratory tests were negative for factor V Leiden, protein C, protein S, antithrombin III, anti β2 glycoprotein 1 IgM and IgG, anti-cardiolipin IgM and IgG; ADAMTS-13 enzyme activity. Immunoglobulins (IgA, IgG, and IgM) were normal, Complement 3 (C3) 86.83 mg/dL and Complement 4 (C4) 12.76 mg/dL, anti-nuclear antibody (ANA) and anti-dsDNA were negative. The results of microbiological tests were all negative including culture test of blood, CSF, endotracheal aspiration; multiplex polymerase chain reaction (PCR) in CSF and blood; HSV real-time PCR in CSF, Japanese encephalitis IgM in blood and CSF; tuberculosis; and nonstructural protein 1 (NS1) antigen of dengue virus.

The fever decreased after 24 hours of starting methylprednisolone. The patient’s condition gradually improved, he had no more seizures, and his consciousness gradually recovered. He was extubated after 10 days of mechanical ventilation. After extubation, he was conscious but he was still weak on the left side of his body with muscle strength of 0/5. Antibiotics and acyclovir were stopped and methylprednisolone was continued for seven days at the same dose, after that, we gradually reduced the dosage and stopped the drug after 17 days of treatment. The patient was discharged on Day 31. Vitamin K antagonist was used after discharge due to femoral and humeral venous thrombosis. He stopped anticoagulation after 3 months of treatment and continued to be monitored until 5 months after discharge. We did not record any new thrombotic events; the patient had a good recovery with normal consciousness and muscle strength of 5/5.

**Figure 3:**
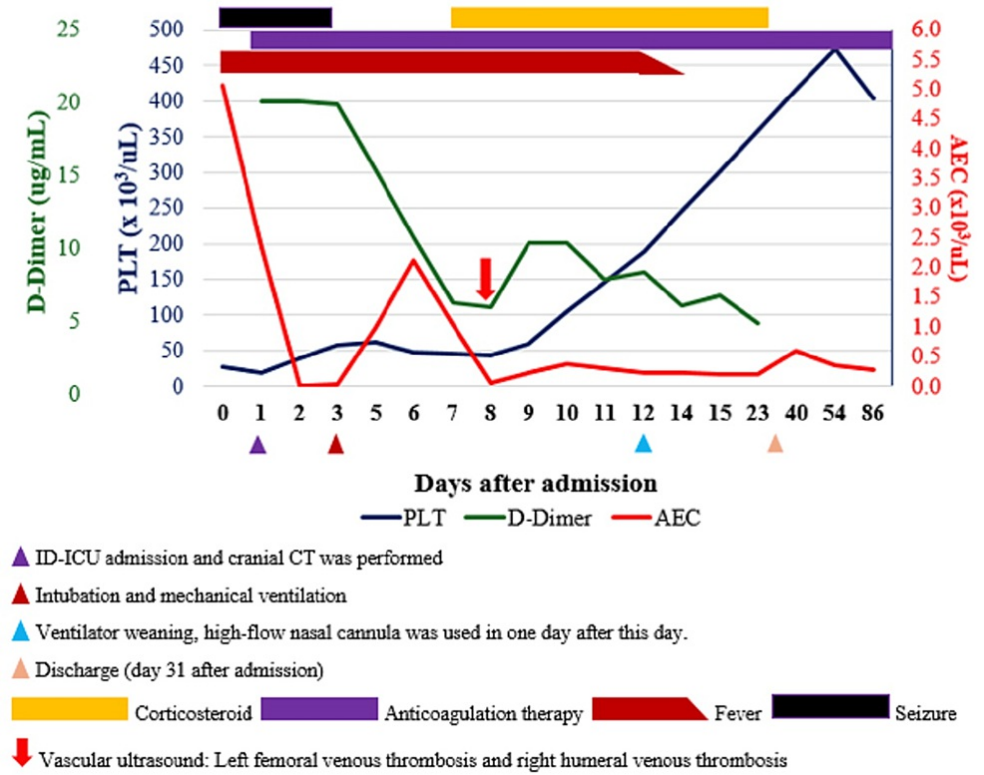
Clinical course and laboratory parameters (absolute eosinophil count, PLT count, and D-dimer). The figure showed that the progression of eosinophil count was inversely correlated with PLT count; eosinophil count gradually decreased to normal after using corticosteroid accompanied with recovery of PLT count, D-dimer gradually decreased after the addition of anticoagulation therapy. AEC: absolute eosinophil count; PLT: platelets

Clinical course and laboratory parameters are shown in Figure 3.

## Discussion

We have presented a case diagnosed with CVT related to HE and complicated by ICH in a child. Thrombocytopenia was a notable clinical manifestation in the patient but we found that eosinophilia is the crucial cause of all his conditions.

The eosinophil performs its functions largely through its mediators, in which the main effects include participating in the body’s defense against helminthic parasites and inflammatory responses in general, regulating homeostasis in certain circumstances, such as wound healing and mammary gland development [3,5]. However, eosinophilic inflammation can cause severe tissue damage in some situations, especially AEC > 1.5x10^3^/μL. There are various causes of eosinophilia/HE, including clonal HE and reactive HE. Reactive or secondary eosinophilia encompasses all medical conditions and disease states (including infections, allergic diseases, primary immunodeficiency, autoimmune diseases, malignancies, drug reactions, etc.) [4]. The patient did not have manifestations of these diseases and did not have risk factors for infections of parasites. He had a positive dengue IgM ELISA that was performed on the 13th day after admission (or the 18th day after the onset of the first symptoms). Based on the epidemiology and clinical features of the patient, it can be confirmed that he could not develop these diseases. Therefore, dengue infection was the most likely cause because of the popularity of the virus in southern Vietnam. Eosinophilia can be found in dengue infection [8]. Dengue-associated stroke has also been reported (ischemic or hemorrhagic stroke) as an infrequent neurological complication of dengue infection [12].

A multicenter, retrospective analysis conducted by Ogbogu et al. showed that the most common signs and symptoms in HE were dermatologic, pulmonary, gastrointestinal, cardiac, and neurologic [13]. Neurologic diseases and thrombotic complications were not common, mainly in adults. Thrombosis is one of the critical features in the diagnosis and treatment of HE-related organ damage (hypereosinophilic syndrome (HES)). Ogbogu et al. pointed out that a quarter of patients with HES develop thromboembolic complications with a mortality rate of 5-10% [14]. Thromboembolic complications caused by HES are particularly difficult to control, especially CVT, this has been proved in a study by Song et al. with a mortality rate of 4/8 cases have been reported [9].

Some main mechanisms of eosinophilia have been proposed on how it can cause venous thrombosis. Direct infiltration of eosinophils and secretion of eosinophil granule proteins such as major basic protein (MBP), eosinophil cationic protein (ECP), eosinophil peroxidase (EPO), and ethylene glycol dinitrate, which can damage the vascular endothelial cells. Eosinophils can directly activate tissue factors, factor VII, factor X, factor XII, and other coagulation factors, which activate the intrinsic coagulation pathway and cause blood hypercoagulability [15,16]. Eosinophils also can directly activate tissue factors and PLT; leukotrienes activate extrinsic coagulation pathways, and activate and aggregate PLT [15]. The release of chemokines can inhibit the production of activated C protein and cohesion with thrombomodulin to prevent anticoagulant activities [15]. Therefore, HE may cause peripheral vasculopathy and also activate coagulation directly, which will lead to the formation of venous thrombosis.

Our patient had both a phenomenon of hypercoagulation and hemorrhage. Thrombosis of the large cerebral sinuses will cause venous congestion and stagnation of circulation, preventing the reabsorption of CSF, contributing to elevated ICP, cerebral edema, ischemic neuronal damage, and hemorrhage from petechia to large hematoma [11,17]. Thrombocytopenia in HE may aggravate the existing bleeding. The mechanism has not been fully understood, however, as described earlier, eosinophilia can cause PLT consumption due to activation and aggregation of PLT. Based on the study of Song et al., there were three cases of CVT with HE reported with thrombocytopenia [9].

Management of CVT complicated by ICH is mainly based on anticoagulation therapy but studies on the use of this therapy in patients with ICH are still very scarce [11]. The simultaneous appearance of CVT and ICH makes the management of CVT more difficult because anticoagulation therapy is a “double-edged sword”, especially in our patient with thrombocytopenia, so the decision to treatment and selection of dosage of an anticoagulant must be very careful. The patient’s cerebral edema during the illness caused us to worry about the progress of ICH if using an anticoagulant dose as the origin. By lowering the anticoagulant dose and monitoring the patient’s condition, we could decrease the risk and stop the progression of ICH. Although current studies suppose this treatment “seemed to be safe” with a low risk of ICH, in fact, it is still associated with a small risk of new ICH with a rate of 3.6-5.4%, eventually 9% [11,18]. The current guidelines of the American Heart Association and European Federation of Neurological Societies allow the use of anticoagulants in the acute phase of CVT, irrespective of ICH, and the recommendation is classified as Class IIA/Level B, but there are no specific recommendations about the timing and the dosage of anticoagulation [19,20]. It is important to regularly assess the risk of progression of intracranial bleeding. Besides anticoagulation therapy, the treatment of HE is very fundamental. Systemic corticosteroid therapy is the first treatment in HE/HES with life-threatening emergencies, like our case [1]. The goal of treatment is to reduce the eosinophil count to less than 1.5x10^3^/µL. The early use of corticosteroids helps to stop pathophysiological mechanisms and prevents new formation of thrombi, especially in the brain, heart, and lungs, which helps improve the prognosis of the patient.

## Conclusions

CVT complicated by ICH with HE in a child is a rare case and management of the condition is a great challenge, requiring careful monitoring of the patient’s clinical progression. In tropical and subtropical countries of the Asia-Pacific and Latin America regions, dengue infection should always be considered as a possible cause because of the wide spectrum of clinical manifestations. This report adds to the varying presentations that a patient with dengue infection can have. Systemic corticosteroid administration and anticoagulant therapy are the cornerstone of treatment, along with measures to control intracranial pressure that will help improve the outcome.
